# Perceived Barriers to Career Progression Among Early-Career Epidemiologists: Report of a Workshop at the 22nd World Congress of Epidemiology

**DOI:** 10.2188/jea.JE20180184

**Published:** 2019-01-05

**Authors:** Hiroyuki Kikuchi, Keisuke Kuwahara, Kosuke Kiyohara, Ester Villalonga-Olives, Naomi Brewer, Abimbola Aman-Oloniyo, Pradeep Aggarwal, María Clara Restrepo-Méndez, Azusa Hara, Masako Kakizaki, Yuka Akiyama, Kazunari Onishi, Kayo Kurotani, Maho Haseda, Shiho Amagasa, Isao Oze

**Affiliations:** 1 Japan Young Epidemiologists Network, Tokyo, Japan; 2 Department of Preventive Medicine and Public Health, Tokyo Medical University, Tokyo, Japan; 3 Teikyo University, Graduate School of Public Health, Tokyo, Japan; 4 Otsuma Women’s University, Tokyo, Japan; 5 Tokyo Women’s Medical University, Tokyo, Japan; 6 Early Career Epidemiologists Committee, International Epidemiological Association, Raleigh, NC, USA; 7 University of Maryland, Baltimore, MD, USA; 8 Harvard T.H. Chan School of Public Health, Boston, MA, USA; 9 University of Otago, Wellington, New Zealand; 10 Massey University, Wellington, New Zealand; 11 Epidemiological Society of Nigeria, Abuja, Nigeria; 12 Swami Rama Himalayan University, Dehradun, Uttarakhand, India; 13 MRC Integrative Epidemiology Unit, Population Health Sciences, University of Bristol, Bristol, UK; 14 Showa Pharmaceutical University, Tokyo, Japan; 15 Department of Public Health, School of Medicine, Fujita Health University, Aichi, Japan; 16 University of Yamanashi, Yamanashi, Japan; 17 St.Luke’s International University, Tokyo, Japan; 18 National Institutes of Biomedical Innovation, Health and Nutrition, Tokyo, Japan; 19 The University of Tokyo, Tokyo, Japan; 20 Aichi Cancer Center Research Institute, Aichi, Japan

Epidemiology has always been considered a key science of public health.^1^ From past to present, scientific evidence arising from high quality epidemiological studies has brought innovation to community practices to control diseases.^2^ Thus, epidemiologists have played an important role in providing high quality evidence for policy making and decisions. To control current and future occurrences of disease, it is important to consider how early-career epidemiologists can continue their careers as epidemiologists.

Generally, early-career researchers face various barriers to their career progression, such as work-life imbalance, insufficient research funds, or job instability.^3^^,^^4^ Identifying these barriers is important when considering beneficial support for career progression.^5^^–^^7^ Past findings suggest area-specific barriers among early-career researchers in endocrinology,^8^ genetics,^9^ and psychology.^10^ However, little is known about barriers faced by early-career epidemiologists.

In August 2017, Japan Young Epidemiology Network (Japan-YEN) and the International Epidemiological Association’s (IEA) Early Career Epidemiologists (ECE) Committee collaboratively arranged a workshop regarding career progression among early-career epidemiologists at the IEA World Congress of Epidemiology (WCE) in Japan. The current manuscript aims to summarize the outcome of the workshop and to describe perceived barriers for early-career epidemiologists in order to determine future support.

## DETAILED WORKSHOP PROCEDURE AT IEA WCE 2017

On August 23^rd^, 2017, the IEA ECE Committee and Japan-Yen collaboratively held a 2-hour session entitled “*Early career epidemiologists - current activity and future development*”. In the latter half of the session, attendees participated in a workshop about career development. The theme of the workshop was “*Barriers and solutions for better career progression among early-career epidemiologists*”. This workshop targeted early-career epidemiologists, and the objectives of the workshop were to identify perceived barriers that prevent career development and to point out any helpful support or solutions to overcome these barriers.

To create discussion groups, attendees were asked to sit at one of twelve tables, depending upon which month of the year they were born. Then, they were asked a few questions in order to elicit their ideas. Two questions, “*What kind of barriers prevent early-career epidemiologist’s career development?*”, and “*Do you have any ideas to overcome the barriers?*” were suggested, and participants wrote down their own ideas on sticky notes. Attendees were instructed to use one sticky note for each idea, and to use yellow ones for perceived barriers and pink for possible solutions.

After completion of individual tasks, group discussions commenced. At first, group members introduced themselves and shared their perceived barriers and ideas for solutions. Then, discussions were held to explore feasible and practical ideas to overcome these barriers. During these discussions, at least one author of the current manuscript joined as a facilitator at each table. After about 30 minutes of discussion, each group shared their ideas with the rest of the attendees.

Finally, a second individual task was introduced, where each attendee considered their “best” idea for career development by asking “*What is your best idea throughout this session?*”. Attendees were instructed to write their ideas down on green sticky notes. In addition, attendees were also asked to write their nationality.

## DATA COLLECTION AND ANALYSIS PROCEDURE

All sticky notes used were collected after the workshop; members of the Japan-YEN transcribed each idea into an electronic database.

Ideas suggested by attendees were classified into several categories as follows: initially, one author developed a draft set of categories by considering word frequency data; thereafter, several authors reviewed the initial draft and developed a second set of categories. Two authors then independently classified all of the ideas into those categories. Inter-coder differences were resolved through discussion. Finally, all Japan-YEN authors confirmed the categories, which were consistent with what attendees discussed in the workshop.

## CHARACTERISTICS OF WORKSHOP PARTICIPANTS

Sixty-seven attendees (25 males, 37.3%) from 17 different countries joined this workshop. The attendees were from Australia, Bangladesh, Brazil, Britain, Canada, China, Netherlands, Ireland, Japan, Kenya, Malaysia, Nigeria, Pakistan, Peru, Portugal, South Korea, and Spain.

## PERCEIVED BARRIERS TO CAREER DEVELOPMENT

In total, 228 perceived barriers were collected from attendees during the individual tasks. As shown in Table 1, these perceived barriers were classified into 10 categories. ‘Lack of funding’ was the most common barrier to career development (*n* = 39, 17.1%). ‘Lack of training opportunities to develop skills’ (*n* = 27, 11.8%) was the second most common barrier. Furthermore, ‘work-life imbalance’ (*n* = 20, 8.8%), ‘lack of research post’ (*n* = 17, 7.5%), ‘lack of network with other researchers’ (*n* = 16, 7.0%), and ‘unavailability of mentorship’ (*n* = 16, 7.0%) were also major barriers suggested by attendees.

**Table 1.** Perceived barriers to career development among workshop attendees

Category	*n*	(%)
Lack of research funds	39	(17.1%)
Lack of good quality training opportunities	27	(11.8%)
Work-life imbalance	20	(8.8%)
Lack of research positions	17	(7.5%)
Lack of network with other researchers	16	(7.0%)
Unavailability of mentorship	16	(7.0%)
Language issues	9	(3.9%)
Lack of scholarship	7	(3.1%)
Difficulty of access to data	6	(2.6%)
Others	71	(31.1%)

Total	228	(100.0%)

## IDEAS FOR POSSIBLE SUPPORT TO OVERCOME BARRIERS

Through both the individual tasks and the group discussions, attendees made 80 suggestions of possible support for better career development. These ideas were classified into five categories, as shown in Table 2. The most consistent suggestion was ‘strengthening the domestic and international network among early-career epidemiologists’ (*n* = 19, 23.8%). In addition, many suggested ‘constructing online courses in epidemiology’ (*n* = 17, 21.3%), ‘support for acquiring research funding’ (*n* = 11, 13.8%), and ‘support for mentors or mentees’ (*n* = 10, 12.5%).

**Table 2.** Ideas for potential solutions suggested by attendees

Category	*n*	(%)
Strengthen networks with other epidemiologists	19	(23.8%)
Online courses in epidemiology	17	(21.3%)
Support for acquiring research funds	11	(13.8%)
Support for mentees and mentors	10	(12.5%)
Others	23	(28.8%)

Total	80	(100.0%)

## SUMMARY OF DISCUSSION AT THE MEETING

This report summarizes perceived barriers to career development among early-career epidemiologists attending a workshop held at the IEA WCE 2017. In addition, this manuscript also summarizes the potential solutions which were suggested by attendees. Many attendees gave ‘lack of research funds’, ‘insufficient research posts’, ‘lack of training opportunities’, and ‘difficulty in balancing work and life’ as major barriers to their career development. To overcome these barriers, attendees suggested ‘building strong networks with other epidemiologists’, ‘constructing online epidemiology courses’, and ‘support for acquiring research funds’ as feasible ideas to combat these barriers.

A systematic review regarding post-doctoral academics showed six key influences on career progression: intrinsic motivation, work–life balance, inclusiveness, work environment, mentorship, and availability of funding.^3^ Considering this review, the perceived barriers among early-career epidemiologists appear to be very similar to those among general post-doctoral academics. In addition, similar barriers have been suggested among researchers in endocrinology,^8^ genetics,^9^ and psychology.^10^ The present study showed that early-career epidemiologists share common barriers with early-career people in other fields, and that early-career epidemiologists around the world share common barriers to their career progression.

‘Lack of research funds’ was the most common barrier among early-career epidemiologists at the workshop. Some examples written were “*Problems in getting funding for projects*” and “*poor budget for research*”. Since other studies have indicated that funding availability is a key issue for career progression among early-career researchers,^3^^,^^11^ this might indicate that any support for acquiring sufficient research funds would be beneficial. Attendees suggested that support for acquiring research funds could include “*webpage for information about grant & funding, guidance for application*” and “*IEA could offer seed grants to encourage ECR in this undertakings*”. In addition, one attendee suggested “*Advice or guidance about grant writing. Paper writing from mentor*”. Support for high quality mentoring would be indirectly beneficial in acquiring research funds, and the resumption of the IEA’s mentoring program^12^ would, therefore, be a valuable international contribution.

Attendees suggested ‘lack of good quality training opportunities’ as the second major barrier for their career. Some wrote: “*lack of effective training opportunities*”, “*limited opportunities to brush up epidemiological skills/technique*”, and “*just a few classes for epidemiology in the university*”. A previous study reported that researchers in developing countries tend to have difficulties in receiving adequate training due to the limited research environment.^13^ Even in developed countries, classes or training in epidemiological research may have different quality by university or institution. As a solution for receiving high quality training, 17 attendees suggested “creating online education courses”, such as: “*online course supported by IEA would be helpful for researchers in developing countries*” and “*e-learning platform to support developing countries*”. Online education systems provide benefits, such as ease of access and free or low-cost content delivery.^14^ These systems offer the potential to enable access to high-quality epidemiological education for students, even in the most underserved regions of the world.^15^^,^^16^ Furthermore, a systematic review showed that online learning for teaching clinical skills is no less effective than traditional means.^17^ A high quality, structured training course for epidemiology may benefit students in many countries. Indeed, some free online courses about epidemiology, such as ActivEpi^18^ (in English) or ICR-WEB^19^ (in Japanese), are already available. Since many attendees suggested online courses as a plausible solution, it may be speculated that these existing resources are not well known or are not widely used by early-career people. Thus, it may be helpful to early-career epidemiologists for an organization like the IEA to provide summary information on their website regarding available online courses, such as course level, target learners, or skill requirement (along with information like the course web address and language).

Another major barrier was ‘work-life imbalance’. Some described “*family issues (spouse, children, and parents)*” and “*delivering babies, especially for female researchers*”. Since early-career researchers are more likely than their more senior colleagues to have time constraints due to child-rearing, they may often face difficulties to balance research and family issues.^20^ Previous research has also identified this issue as a major barrier, especially among female researchers.^21^ Imbalance between work and life triggers low job satisfaction and burnout.^22^^,^^23^ To develop effective measures for achieving better work-life balance to be developed, the fact that work-life balance is a major issue among early-career epidemiologists should be publicized more widely. Also, as shown in some ideas from attendees, online mentorship or learning opportunities may be beneficial for early-career epidemiologists because accessing online content is very flexible in terms of time and location.

Finally, this workshop clarified the importance of an international network between early-career epidemiologists. Attendees perceived ‘lack of network’ as a barrier by addressing points, such as: “*lack of international communication*”, “*lack of references and networks to ask for help with specific topics for different areas of research*”, and “*lacking of the collaboration between the epidemiologists inside the country*”. In addition, they were eager to create more collaboration internationally, as seen in the comments: “*strong ties between early career researchers as well as experts in other areas*” or “*young Epi connect and support each other worldwide*”. International networking has grown in other fields.^10^^,^^24^^,^^25^ Since regional associations among early-career epidemiologists in places like Japan and Western Europe have been growing recently, it is time to connect these regional networks globally to develop a wider international network, preferably under the leadership of the IEA.

There are some limitations of this study that should be considered. First, detailed information regarding attendees was unavailable, and perceived barriers may differ by gender, country of birth, position, or past experience. Second, it cannot be said that workshop attendees adequately represented the whole of early-career epidemiologists. Since all attendees could afford to join this international conference, it is possible that they have a better work environment than those who could not join. Large-scale future research that systematically includes early-career epidemiologists with various backgrounds is needed to further explore detailed barriers and solutions. Several strengths were also indicated for this study. First, the workshop included more than 60 early-career epidemiologists from 17 different countries, so it may reflect ideas from across the globe. In addition, to the best of our knowledge, this is the first study to attempt to identify career progression barriers among early-career epidemiologists.

## CONCLUSION

Early-career epidemiologists who attended a workshop at the World Congress of Epidemiology in 2017 described “lack of funding”, “lack of good quality training”, “imbalance of work and life”, and “lack of network” as frequent and internationally consistent barriers to career development. Effective countermeasures to these barriers are necessary for career progression.
